# Global survey of consumer organizations advocating for safer nicotine products

**DOI:** 10.1002/puh2.58

**Published:** 2023-01-19

**Authors:** Tomasz Jerzyński, Jessica Harding, Gerry V. Stimson

**Affiliations:** ^1^ Robert Zajonc Institute for Social Studies University of Warsaw Warsaw Poland; ^2^ Knowledge Action Change London UK; ^3^ Department of Social Science and Medicine Imperial College London London UK

**Keywords:** consumer advocacy, organizations, safer nicotine products, tobacco harm reduction

## Abstract

**Introduction:**

The study investigated the role and activities of organizations that advocate for the adoption of and access to safer nicotine products (SNPs), such as nicotine vaping products (e‐cigarettes), Swedish‐style snus, nontobacco nicotine pouches, and heated tobacco products, as safer alternatives to combustible cigarettes and other high‐risk tobacco products, following a harm reduction approach. The aim was to map the number and locations of nicotine consumer organizations globally and describe their history, legal status, membership, structure, objectives, working methods and activities, and funding.

**Methods:**

We identified active organizations through the use of existing networks and referrals. All identified organizations were contacted and asked to fill in an online self‐completion survey through the representatives of the organizations. The data collected were cleaned and anonymized. Categorization and analysis of variable distributions were carried out. Responses to open–ended questions were analyzed qualitatively.

**Results:**

A total of 52 active organizations were identified: 13 in Latin America, 8 in Africa, 24 in Europe, 5 in the Asia‐Pacific region, and 2 in North America. Most were established from 2016 onward, and 39 were legally incorporated. Their reported objectives were to raise awareness about SNP, promote rights to access SNP, and advocate for a legal and regulatory environment in which SNPs are available. They are small organizations: Most are operated with volunteers, with only 7 having any contracted or paid staff, and only 13 persons globally with a paid position. A total of 31 organizations had not received any funding support. The total global funding for all organizations was US$ 309,810. None reported receiving funding from tobacco or pharmaceutical companies. All pointed to important achievements in the public debate about SNP and tobacco harm reduction.

**Conclusion:**

The organizations are run by enthusiastic individuals, most of whom have successfully quit smoking with the help of SNP. Organizations depend on the input of a small number of core workers, all organizations are under‐resourced and potentially fragile, and yet, they report significant activity and success. The challenge for these groups is to gain recognition at national and international level as legitimate stakeholders in the development of tobacco control policy with respect to safer alternatives to smoking.

## BACKGROUND

The combustion of tobacco is the main cause of tobacco‐related morbidity and mortality [1]. Tobacco harm reduction using safer nicotine products (SNPs) offers options for smokers who wish to switch away from smoking but who are unable or unwilling, to stop using nicotine [2]. SNPs are noncombustible (there is no burning of tobacco) and are substitutes for cigarettes and harmful oral tobacco products (such as tobacco mixes like gutka, khaini, paan masala, kattha, zarda, and chuna, which can contain, for example, slaked lime, areca nut, and spices). They include nicotine vaping products (e‐cigarettes), heated tobacco products, Swedish‐style snus (a pasteurized oral tobacco), and nontobacco nicotine pouches. There is also a wide range of products classified as nicotine replacement therapy. Their availability is mainly limited to pharmacies and includes nicotine patches, nicotine chewing gum, and nicotine inhalers.

Most SNPs (except Swedish snus) have only become available on the market in the last 15 years. These include e‐cigarettes (introduced around 2007), heated tobacco products (available around 2014) [3], and nontobacco nicotine pouches (introduced around 2018) [4]. Despite their relatively recent introduction, it is estimated that there were 68 million e‐cigarette users worldwide by 2020 [5], and the total population using e‐cigarettes, snus, or heated tobacco products may be around 100 million [6]. The number of users of nicotine e‐cigarettes had risen to 82 million in 2021 [7]. The number using SNPs is still small in comparison with the World Health Organization's (WHO) estimate of 1 billion smokers globally [8].

The dynamics of this uptake of SNPs has been little explored. It involves multiple factors, including consumer interest, product development and availability, regulatory frameworks, government policy, and activities of public health organizations. Tobacco harm reduction and the use of SNPs has become a hotly contested arena in public health and tobacco control. A wide range of groups has been advocating against the use of SNPs, including the WHO, international philanthropic organizations, and nongovernmental organizations (NGOs). Substantial funding support has been provided, mainly by the Bloomberg Philanthropies to WHO [9]. The scarcity of funds from Member States has led to WHO increasingly relying on money from private entities to fund global tobacco control activities, primarily from the Bill and Melinda Gates Foundation and the Bloomberg Philanthropies [10]. The Bloomberg Philanthropies plays a major role in international tobacco control, with a priority on prevention for young people, including investing in campaigns against e‐cigarettes [11, 12].

What role do consumers play in this dynamic? The majority of SNP users are probably unaware of the political, policy, and regulatory issues surrounding these products. However, there has been an observed growth of small grassroots organizations, established and run by nicotine consumers, which advocate for SNPs. Most of these organizations have emerged over the last 8 years or so. These groups are sociologically interesting as examples of loosely organized special interest and activist groups driven primarily by personal experiences.

There has been little documentation of the history of nicotine consumer advocacy. It arose mainly in response to legal or regulatory threats to the availability and accessibility of safer products. In Europe, the main impetus came with the publication of the draft EU Tobacco Products Directive in 2014 (2014/40/EU) [13]. Across Europe, numerous e‐cigarette users emailed their members of the European Parliament, explaining how in their view vaping had saved their lives by enabling them to stop smoking, and demanding that vaping remained a consumer product, rather than being banned or regulated as a medical product [14]. Objections to the EU TPD were also expressed through online forums and on social media.

Initially, there were few formal organizations advocating for SNP. The Consumer Advocates for Smoke‐free Alternatives Association (CASAA) in the USA, founded in 2009, was one of the earliest organizations. Many small consumer organizations and associations began to emerge from around 2015 onward, initially mainly in Europe and Australasia. Little has been written about these organizations and contact with them indicates that many are loosely organized, run by volunteers, with little formal makeup, and with no previous experience of advocacy. A few studies have purported to show that they are “fronts” for tobacco companies, with the implication that they are funded by tobacco companies or that these companies work closely with them. Such studies have employed methods like mapping Twitter activity [15] or analyzing media reports and have not utilized other sources of primary data [16, 17].

From a social policy perspective, there are interesting questions to ask about the visibility and impact of such small grassroots organizations, in the context of the dynamics and debate with more substantially funded organizations that promote contrary views. In this context, the present study aims: (a) to map the number and location of nicotine consumer organizations globally, regionally, and nationally, and by language; (b) to describe their history, legal status, membership, structure, objectives, working methods and activities, links, and funding; and (c) to record self‐reported achievements and impacts and the challenges they face. This is the first study of this kind.

## METHODS

### Study design and population

This is a descriptive cross‐sectional study employing an online survey conducted between March 7 and May 5, 2022. Respondents were nicotine consumer organizations. In this study, we defined these organizations as groups with a named identity and organized by consumers. They (a) are concerned primarily with SNPs, that is, noncombustible products, such as nicotine e‐cigarettes, Swedish‐style snus, nicotine pouches, or heated tobacco products; (b) have a within‐country, national, or regional focus; (c) undertake advocacy and awareness raising, such as by using media and social media, organizing meetings, and contacting parliamentarians; and (d) are not primarily a trade or product association—such as associations of manufacturers, distributors, or retailers of SNP. The study excluded all organizations that were primarily organized and run by professionals.

### Study instrument and variables

A questionnaire was developed, which included questions that revolved around the organization: history, structure, membership, resources, funding, and activities. The questionnaire was revised after a pilot was done and after consultation with regional research collaborators. The questionnaire included questions with multiple choices and open–ended questions allowing narrative responses.

### Data collection and analysis

Organizations were identified through an extensive network, in all global regions of the world, consisting of regional nicotine advocacy umbrella groups in Africa, Latin America, North America, Europe, and Asia‐Pacific. Snowballing was done through the same organizations. All identified organizations were contacted and asked to fill in the online self‐completion survey. Respondents were asked to participate in the study through a personalized invitation letter from the researchers. Each organizational respondent had a unique access code to the questionnaire. Data analysis was done through descriptive statistics and qualitative analysis of responses to open–ended questions.

### Ethics statement

Ethics permission was granted by the Robert Zajonc Institute for Social Studies, University of Warsaw, Poland, application: 19/21. Participation in the study was voluntary, and there were no financial incentives provided to be a part of the study.

## RESULTS

The study team was able to identify 67 organizations globally. During the data gathering, it was found that 13 organizations were no longer operational and, thus, were excluded. It is possible that there are other existing organizations that may not have been identified and reached. The effective sample was 54, and replies were obtained from 52; thus, a very high response rate was achieved. This high response rate is attributed to the organization, Knowledge Action Change (K A C), which managed this research study and is known and trusted by many of these responding organizations. K A C is a private sector public health agency that runs the Global State of Tobacco Harm Reduction project and the Tobacco Harm Reduction Scholarship Programme. Most of the questionnaire responses were completed by the senior leadership of each organization that was contacted, such as the chair, president, trustee, or founder. Data were categorized under pre‐identified themes as follows: (a) nature of the organization, (b) emergence of the consumer organization, (c) how the consumer organization was organized, (d) products being advocated, (e) organizational objectives, (f) working methods, (g) organizational resources, and (h) organizational achievements and prospects.

### Nature of the organizations

All organizations operated across the whole or most of a country (national level organizations), with some organizations also operating in neighboring or other countries with the same language. In some countries, these organizations are working in potentially adverse and hostile contexts. Many of these organizations are small and often run by one or two people. In this article, names of organizations were anonymized, except where information is in the public domain, in order to avoid the possibility of individuals being identified through the name of the organization and the country location.

**FIGURE 1:**
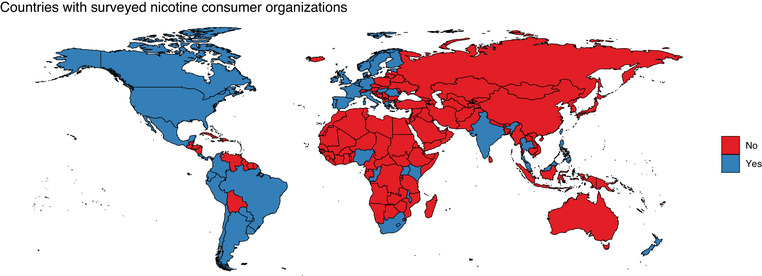
Countries with surveyed nicotine consumer organizations

Many of these organizations were members of four regional umbrella organizations covering Latin America, Africa, Europe, and Asia‐Pacific. There was a wide geographical presence with 13 organizations operating in Latin America, 8 in Africa, 24 in Europe, 5 in the Asia‐Pacific region, and 2 in North America (see Figure 1). No organizations in Eastern Europe, Central Asia, or the Middle East were identified despite extensive contacts in these regions. The groups operated in 27 languages, which were Bahasa Melayu, Chichewa, Danish, Dutch, English, Estonian, Filipino, Finnish, French, German, Greek, Hindi, Hungarian, Italian, Kiswahili, Luganda, Mandarin, Nigerian Pidgin English, Norwegian, Portuguese, Romanian, Spanish, Swahili, Swedish, Thai, Tumbuka, and Turkish.

### Emergence of consumer organizations

Most of the organizations had a very short history, with the earliest being established in 2009 and 36 having started from 2016 onward. Most started informally as small groups of smokers who had switched to vaping or other SNP and then developed into grassroots organizations. As provided by one such group:

[organization name] is a community that started in mid‐2017. The origin was a testimonial portal on Facebook for [country name] e‐cig users to share their experiences.

All organizations were started by smokers who had switched to SNP, rather than being founded by professionals. Personal and organizational trajectories exemplify how the founders moved from personal interest to organizational engagement. It is likely that most did not set out to be advocates or to set up organizations. The accidental route into advocacy was typified by the head of one organization who remarked:

I didn't realize I was doing advocacy, I thought I was just harassing politicians!

One of the respondents described her path to advocacy:

I came to consumer advocacy as someone who smoked for 26 years who could only stop smoking when I found vaping, back in 2012. I got actively involved when the European Legislators tried to introduce regulations which would have prevented smokers from taking up vaping. I could not believe that the miracle cure for smoking was going to be taken away. Along with thousands of other vapers, I sent emails to representatives in Europe in 2012. We were successful in preventing the EU from taking vaping products off the consumer market.

### How consumer organizations were organized

The informal, grassroots nature of how these organizations started and developed is reflected in the wide range of formalization processes of these organizations. Almost a quarter of them (12) were informal groups or associations with no legal incorporation, for example, a group of associates or friends, or a Facebook or WhatsApp group. A total of 39 were legally incorporated as a NGO, not‐for profit, charity, foundation, or organization of a similar nature. Most of these groups with legal incorporation (30 of them) had gained this status fairly recently (since 2015). A few were unable to incorporate due to the legal status of e‐cigarettes:

Since [country name] still bans the import, manufacturing and distribution of vape items, it is near impossible for us to register as a legal entity.

The formal organizational status is reflected in the way in which organizations are actually run, with 14 having no formal structure, 14 having an informal structure, and 24 having a formal governing board, for example, having a Board of Trustees, directors or company owners as shown by the following quote:

We have an elected board, with president, vice‐president, treasurer, two elected members, and two deputies. We are a non‐profit consumer organization, that speaks on behalf of the [country name] vaping consumers.

Not all the organizations have a formal membership, and the nature of relationships between the organization and those it seeks to represent varied widely, for example:

We don't have formal membership, but ask for contact information for those who want to register with us.

The surveyed groups can be described as either loosely organized organizations (mainly with a social media presence) that had followers (up to a maximum of 300,000 in one example) or supporters, and those which had a more formal provision for individuals to join as members or associates, with or without a joining or membership fee. Most organizations reported an increase (21 organizations) or unchanged (20 organizations) number of members in the last 12 months. Ten organizations reported a decrease in the number of their members.

### Products being advocated

The focus of activity is reflected in the names of the organizations. Thirty six had some reference to vaping or e‐cigarettes in their name, 22 had a generic name, such as referring to nicotine, tobacco harm reduction, or safer alternatives to smoking, and 2 directly referred to snus. Seven organizations reported advocating for all or most SNPs. Ten organizations indicated that in addition to their core focus, they also support all other tobacco harm reduction products. However, most of them are focused on a specific SNP. Twenty eight indicated advocating mainly for e‐cigarettes, and an additional 21 organizations listed e‐cigarettes as one of their items of interest (see Table 1).

**TABLE 1:** The range of products advocated for by the organizations surveyed

e‐cigarettes	Snus	Nicotine pouches	Heated tobacco products	Nicotine replacement therapy	All/most safer nicotine products	N
					X	8
	X					1
	X	X				2
X						28
X			X			2
X		X	X			2
X	X	X				4
X	X	X		X		1
X	X	X	X			2
X	X	X	X	X		2
**Sum**						**52**

### Organizational objectives

The objectives of most organizations were to raise awareness amongst smokers, the public, government, and the media about safer alternatives to smoking, and to advocate for a legal and regulatory environment in which products are available, as shown by the following quote:

[organization name] is a consumer rights association involved in improving products quality, information & choice (standards, regulations, certifications), defending vaping products consumer's rights, and helping authorities & health professionals to better know the products, how they are used/best used, and their contribution to reducing smoking.

Other organizations focus on the provision of information to the public, as shown by the following:

Our tasks are to provide updated and accurate information on non‐combustible nicotine consuming products, to contact media, develop social networks and try talking to legislators and MDs. The aim is to advance a risk proportionate regulation of the devices in [country name], as currently their commercialization and imports are illegal.

“Rights” were central in many of the narratives of the organizations. This was a common theme presented by a number of them, as shown by the following quotes:

[organization name] The main purpose of the Association is to protect and defend the rights of users of personal vaporizers who are of legal age, to use and acquire their devices freely in order to minimize the health risks caused by tobacco consumption, or as a simple recreational activity, based on the free development of their personality as established in the Political Constitution […].

[organization name] is an organization created to stand for the rights of every single person who is of legal age to use e‐liquid vaporizers to quit tobacco […] We fight for our right to vape, being aware that for many of us vaping has saved our lives, based on the principle that “Harm Reduction is a Human Right”.

In terms of actions, some of the same organizations presented their strategies and approaches to address issues as follows:

[…] we try to defend vaping from the obstruction, mainly amongst the regulatory and media frameworks, of those groups who place their own economic benefits above the clear advantages that these devices contribute to Public Health, due to their enormous potential on reducing the damages caused by smoking.

[…] exposing anti THR crusaders’ lies about snus and nicotine, documenting it and showing it to the public.

### Working methods

Many of the organizations share common approaches to work. The main activity of almost all organizations is to provide information to their supporters. More than 40 organizations reported networking with parliamentarians or government officials and establishing and maintaining international contacts. More than three‐quarters were involved in raising the profile of SNPs on social media and discussing regulatory and policy issues on these platforms (see Table 2).

**TABLE 2:** Main and subsidiary activities

	Main		Subsidiary	
What are the main (and subsidiary) things that you do?	*N*	%	*N*	%
Provide information to supporters	50	∼96	2	∼4
Make contact with journalists	32	∼62	18	∼35
Raise profile of Safer Nicotine Products on social media	41	∼79	10	∼19
Discuss regulatory and policy issues on social media	44	∼85	8	∼15
Produce materials for broadcast streaming in audio or video	20	∼38	29	∼56
Run campaigns	24	∼46	25	∼48
Make contact with parliamentarians/government officials	44	∼85	6	∼12
Make and maintain international contacts	42	∼81	9	∼17
Organize meetings	24	∼46	25	∼48
Provide information such as newsletters and/or websites	39	∼75	12	∼23
Responding to consultations	37	∼71	13	∼25

Representatives of these organizations mentioned activities, such as pursuing risk‐proportionate regulation for SNP separate from combustible products, conducting petitions and surveys, helping with defending the right to health, and “[giving] users legal means to fight prohibitions […].” Organizations also kept members up‐to‐date on new developments in nicotine science and research, interacted with academics, participated in maintaining product quality (standards) and certification, and recruited users, entrepreneurs, and activists to achieve a multi‐stakeholder community.

Organizations are in a continuous state of flux. Thirteen that were identified were no longer operating thus no survey information was gathered. Twenty six organizations reported engaging in more or many more activities than the previous year, 11 were doing about the same, and 15 were doing less or far less than the previous year.

The main example of organizational visibility was a social media presence. Only one organization reported no online presence. Most of the organizations had a website (44 organizations) and a presence on social media, such as a Facebook group (21 organizations), Facebook page (45 organizations), Instagram (20 organizations), Twitter (42 organizations), and other social media (3 organizations). Groups formed on instant messaging platforms were also popular (WhatsApp, Messenger, Signal, Skype, etc.) (11 organizations). Many organizations have created and shared content on streaming services (YouTube, Vimeo, etc.) and/or audio podcasts (20 organizations). Only four of them used older platforms, such as forums, discussion, or chat list. Given the various ways in which social media presence may be measured, it is therefore difficult to provide a good estimate of organizational reach; however, 30 organizations estimated their reach to be between 100 and 10,000 followers and 16 organizations at over 10,000 and up to 300,000 followers, respectively.

### Organizational resources

Crucial to organizational activity and reach are the operational resources available to the organization in terms of staff and funding for their activities. The number of staff is generally small. The vast majority (42 organizations) operated with volunteers, with only 7 having any contracted or paid staff. The maximum number of staff was three (for two organizations)—another two organizations had two employees each, and three organizations had one person each. Taking the survey sample as a whole, this equates to only 13 people in paid positions among all these organizations surveyed. These 13 paid staff worked a total of 158 h a week between them. For those organizations with paid staff (7 organizations), the number of hours worked per‐week‐per‐person ranged from 5 to 40 with a mean of 10 h. The total number of staff, whether paid or unpaid, ranged from 10 to 30 with a mean of 13. This equates to 122 persons with a formal role in all nicotine consumer organizations.

The lack of operational resources is reflected in the low level of funding. Thirty one organizations had not received any funding support. For the 21 organizations with some funding, this ranged from US$ 250 to US$ 173,500 for the last full year, with a mean of US$ 14,753 per funded organization. A total of 16 organizations had an income of between US$ 250 and US$ 10,000 and 5 organizations of over US$ 10,000. Overall, for the last full year, the total funding for all organizations surveyed was only US$ 309,810. The most common sources of funding were from donations (10 organizations) and membership fees (9 organizations); three organizations received donations from vaping companies. None had funding from tobacco or pharmaceutical companies. One commented that:

our statutes don't admit any interference, economic or otherwise, from industry: be it big tobacco, pharmaceutical, philanthropist, vaping sector or tobacco control.

The lack of resources is reflected in the views of the organizations about what they would require to operate more effectively, including more volunteers (40 organizations), funding (32 organizations), skills training (24 organizations), resources such as briefing papers and documents (24 organizations), paid staff (20 organizations) and own language resources (17 organizations).

### Organizational achievements and prospects

Despite the poor organizational resource and funding base, all organizations were able to enumerate important achievements in the past 12 months. For example, engaging the media, gaining print, and broadcast media coverage, making submissions to consultations by government and regulatory and advisory organizations, participation in public hearings, hosting webinars and face‐to‐face meetings, contacting parliamentarians, helping new organizations to launch, running social media campaigns, organizing protests, pursuing legal challenges, developing contacts with government ministries, and contributing to the development of national e‐cigarette standards. Many responses were written lengthily showcasing what they have done as shown by the following:

We have published articles on our website about research and news on e‐cigarettes and the ongoing reform of the Tobacco Act in [country name]. We have also issued official statements on the latter to the Ministry of Social Affairs and Health and sent a letter to MPs calling for the harm reduction approach to be taken into account in the forthcoming Tobacco Act. We have also contacted [country name] MEPs about the Europe's Beating Cancer Plan. We participated in a survey of consumers of nicotine products. In addition, we have repeatedly sent corrections to various health authorities and newspapers regarding the misinformation about e‐cigarettes and snus.

The main obstacle reported by all surveyed organizations was the lack of funds and resources. Most of them also pointed to a lack of volunteers and community engagement, a lack of active and mobilized participation from e‐cigarette users and consumer membership, and difficulties in reaching interested individuals. Frequently reported were negative attitudes from most of the media and health professionals, doctors (one respondent described them as “greedy and corrupt”), and individuals from tobacco control organizations.

Resistance to THR in government circles, such as negative attitudes from Ministries of Health or inappropriate or misguided regulations, was reported as a significant obstacle. Difficulties were noted by some regarding access to influential politicians and public health groups, and corruption and political instability. Many respondents pointed to the scientific ignorance displayed by many anti‐THR organizations. They highlighted harmful disinformation efforts of well‐funded groups (e.g., those funded by the Bloomberg Philanthropies). Opposition from WHO and public health organizations was also indicated, as well as misleading news and unreliable science leading to e‐cigarettes not being recognized as a harm reduction tool.

Some respondents asserted that negative policies have caused a decline in e‐cigarette users, and that, in some highly restrictive settings, those vendors who have found a foothold in the gray market do not feel strongly about the need to support the movement for better regulation or legalization. Apathy in the market, lockdowns due to COVID‐19, and societal moral and religious biases were also mentioned. For many organizations, the main challenges that were reported regarding SNPs in their respective country in the coming 12 months were as follows: possible decreases in access to SNP, including potential sale bans (20 organizations), potential taxation (30 organizations), potential restrictions (e.g., flavor bans, nicotine limits—34 organizations), and potential bans on using SNP in public places (21 organizations).

A lack of resources was a major impediment. “I've not enough time to properly manage some real campaigns, I do this in my spare time” and “we have regular jobs, so the main obstacle is finding time” were typical comments. Some noted the lack of interest from the wider community, a lack of funds, reliance on volunteers in the context of negative information about SNP from government and health authorities, negative media, resistance to tobacco harm reduction, biased media and a lack of support from experts. Much of their time was spent in countering misinformation, for example:

Incomprehensible opposition, a large part, if not most of our time, is used countering fake news, fake science, and anti‐harm reduction, when most if not all should be used improving product information and quality, promoting vaping as an alternative to smoking, and improving accessibility hand‐in‐hand with authorities.

We also face frequent ad hominem attacks from Bloomberg‐funded NGOs run by professional and well‐funded lobbying activists […] those opposing us have many more resources.

## DISCUSSION

The last 15 years have seen a proliferation of SNPs that provide opportunities for smokers to switch away from combustible products [2, 3, 4] and which make tobacco harm reduction a practical alternative for many of the world's smokers and users of harmful oral tobacco products (mainly South Asian–type tobacco mixed with other products). In that period, there has been rapid growth in the number of SNP users globally [5, 8]. The individual and public health opportunities of SNP have been met with a mixed response in the field of tobacco control with some pointing to past failed experience with low tar “light” cigarettes [18, 19] and fear that new products are a tool for manufacturers to continue to provide profitable addictive products [20]. There has been a wide range of regulatory responses, with 36 countries banning the sale of e‐cigarettes, 75 regulating them, and 85 having no specific laws or regulations; a total of 39 countries ban the sale of snus, and 13 ban the sale of heated tobacco products [6]. Contrasting responses are illustrated by the United Kingdom, which has a positive approach to e‐cigarettes, and Australia, which has a de facto ban with products only available on prescription [21].

Paralleling the rise in the use of SNP has been the development of nicotine consumer advocacy. Consumer advocates are usually ex‐smokers who stopped smoking themselves, by using safer alternatives to smoking. Many advocates operate individually, mainly on social media, and are not part of a formal group. Others have come together and organized as formal organizations. There has been speculation about these organizations, including that they are linked with or funded by tobacco companies [15, 16].

This is the first study to map these advocacy organizations and report on how they actually operate. We gained information from 52 organizations. It is possible that not all the organizations currently operating were identified and reached despite the exhaustive attempts to locate consumer advocacy organizations using global contacts.

All of the 52 consumer groups are grassroots advocacy organizations that developed organically from small groups of contacts, including through social media. Consumer organizations reported that they advocate for the right of access to SNP, for better regulation and consumer protection, oppose bans that would deny access to these products, counter misinformation, and engage the public, scientists, and parliamentarians. Some of these organizations are part of wider umbrella organizations in Asia‐Pacific, Africa, Latin America, and Europe.

These organizations resemble and function similarly to other issue‐based health and social advocacy organizations [22]. One area where they differ is in the field of management, as most of these small organizations have no professional staff, are led by volunteers, have minimal or nonexistent organizational budgets, and little prior experience in public affairs and advocacy. These organizations are under‐resourced and are challenged by sustainability issues due to a small number of core workers and dependence on volunteers, with only seven of the organizations having contracted or paid staff. The paucity of resources is quite pronounced with only 13 persons from among all these organizations having a paid role.

Sustainability of these organizations has been raised as one of the main concerns that have come out of the data. Most of the organizations do not receive any form of funding support. The total global funding for all nicotine consumer organizations was US$ 309,810 in the previous year. This reflects the fragility of the existence of these organizations when it comes to resources.

There is a quandary for these organizations regarding how to raise operational funding. This is particularly true for new organizations with no or minimal staff as they have little or no fundraising experience or contacts with donors. There is also the challenge of the ever‐pervasive assumption that they are close to, or are being funded by, the tobacco industry [23, 24]. It is on these grounds that some organizations have been excluded from national consultations and denied observer status at the Conference of the Parties to the WHO Framework Convention on Tobacco Control at the international level [25]. Based on this survey, none of the organizations indicated receiving any funding from tobacco (or pharmaceutical) companies, and many expressed their antipathy to seeking or accepting such funding.

The stature of these organizations is illustrated by the amount of support, resources, and funding that they have access to, compared to other organizations that campaign within the same space against tobacco harm reduction. Power disparities can be exemplified by, for example, the Campaign for Tobacco Free Kids that received US$ 160 million from the Bloomberg Philanthropies to campaign against flavors in nicotine vapes. The University of Bath, which set up the Tobacco Tactics and Tobacco Control Research Group, has received US$ 20 million from the Bloomberg Philanthropies [9]. Some of the funding is used to criticize tobacco harm reduction groups and individuals and to allege that they have links with tobacco companies [6]. The power and clout of the Bloomberg Philanthropies permeate the walls of the WHO through its cumulative funding amounting to 1 billion dollars. These dynamics between Bloomberg and the WHO, both of which have expressed outright antipathy toward tobacco harm reduction and SNP, do not create a space for discussion and debate among the players including the organizations studied [26]. In fact, such disparity in philosophically driven funding further drowns voices as funds are channeled to support more like‐minded organizations. For example, the Campaign for Tobacco Free Kids funds many parent groups active in campaigns against vaping [27]. Bloomberg thus uses its resource advantage to influence international and national tobacco and vaping policies [28].

There is an increasing recognition of the importance of including consumer and patient views in the development, crafting, and implementation of health policies. As a classic example, during the time of the HIV/AIDS crisis, stakeholders coalesced around the slogan “nothing about us without us,” which emphasized that no policy decisions should be made without the participation of those affected by policy. This also came into prominence within disability activism in the 1990s [29]. The idea stresses that people affected by these issues have important expertise to offer, that they are the ones whose lives are affected by government decisions, and that they are the ones to implement changes in health behavior. The important role of advocacy organizations is now recognized in many countries, by international donors, and by UN organizations [30]. This is exemplified by the inclusion of provisions and providing space for inputs from NGOs and the civil society. Sadly, this ethos of engagement has not yet permeated the space of international and national tobacco control and policy‐making.

## CONCLUSION

There is clearly a major imbalance in resources available to groups that are for or against tobacco harm reduction, thus muffling the debate in this space. This study has shown that consumer organizations that advocate for SNPs are run by enthusiastic individuals, most of whom have successfully quit smoking with the help of SNP. These organizations depend on the goodwill and input of a small number of core workers, they are extremely under‐resourced and potentially fragile, and yet they report significant activity and successes in promoting tobacco harm reduction. The immediate challenge for nicotine consumer organizations is how to move from a low and fragile start‐up phase to become better resourced. The further challenge is to gain recognition at a national and international levels as legitimate stakeholders in the development of tobacco control policy with respect to safer alternatives to smoking.

## AUTHOR CONTRIBUTIONS

Tomasz Jerzyński: *Conceptualization; data curation; formal analysis; investigation; methodology; project administration; resources; software; supervision; validation; visualization; writing—original draft; writing—review and editing*. Jessica Harding: *Conceptualization; project administration; resources; writing—review and editing*. Gerry V. Stimson: *Conceptualization; data curation; funding acquisition; investigation; methodology; project administration; writing—original draft; writing—review and editing*.

## CONFLICT OF INTEREST

All authors work for an organization (Knowledge Action Change) that focuses on tobacco harm reduction. The Foundation for a Smoke‐Free World had no role in the planning or execution of this study, data analysis, or publication of results. The contents, selection, and presentation of facts, as well as any opinions expressed are the sole responsibility of the authors and under no circumstances shall be regarded as reflecting the positions of the Foundation for a Smoke‐Free World.

## Supporting information

Questionnaire

Invitation Letter

## Data Availability

The data that support the findings of this study are available on request from the corresponding author. The data are not publicly available due to privacy or ethical restrictions. A significant part of the data contains open text responses which, by their nature, cannot be fully anonymized.
