# Supplementary material for: Global survey of consumer organizations advocating for safer nicotine products
Source: Public Health Chall. 2023 Jan 19;2(1):e58. doi: 10.1002/puh2.58 (PMC12039648; doi:10.1002/puh2.58)
Supplement: Supplementary file 1 — Questionnaire [file PUH2-2-e58-s002.docx]

# Questionnaire of the Survey of Nicotine Consumer Organisations

Dear respondent,

Thank you for joining our survey. Here are some tips that should make filling it out easier for you.

- If your organisation is a member of a larger **association,** please refer only to your division or membership group.
- If you do not want to, or cannot enter exact numbers, enter **approximate** values or **ranges**.
- It is important to answer every question even if the answer is NO – negative answers are just as valuable as positive ones.

If you have any questions or need help completing the questionnaire, please ask {ADMINEMAIL}.

There are 41 questions in this survey.

## Agreement

General information and agreements.

### [Q1] Please give the full name of your organisation and your role within it.

### [Q2] Consent to participate

Please read all the 6 sentences below. If you agree with all of them, please select the box.
It is necessary to agree with all of them in order to participate in the survey.

- - 1. I agree to participate in the study voluntarily and without financial or other reward.
    2. I have been informed that no personal information of mine will be collected or published.
    3. I have been informed that my contact information will be available only to the study authors.
    4. I have been assured that my individual answers will not be publicly available.
    5. I have been assured that the results of the survey will be published in articles and reports in anonymized, aggregated form only.
    6. I am aware that I may withdraw my participation at any time.

If you agree with all 6 sentences above, check the box.

- I have understood, acknowledged, and agree with each of the 6 sentences above.

## Start and status

### [Q3] When did your organisation start? This might be before its legal incorporation, if relevant.

Your answer must be between 2000 and 2022.

Only an integer value may be entered in this field.

Please write a year [_][_][_][_]

### [Q3a] Please comment or clarify if needed.

Please write your answer here:

### [Q4] Please tick the box that best describes what kind of organisation you are.

Please choose only one of the following:

1. We are an informal group or association with no legal incorporation, e.g. a group of associates or friends, or a Facebook or WhatsApp group.
2. We are legally incorporated as a non-governmental organisation, not-for profit, charity, foundation or similar.
3. We are legally incorporated as a for-profit organisation, for example a trading company.

### [G4a] Comments: please add any further information that will help us understand the type of organisation you are.

Please write your answer here:

### [Q5] Is your organisation in the process of incorporation?

Only answer this question if the following conditions are met: ([Q4](file:///C:\index.php\questionAdministration\view\surveyid\416974\gid\2\qid\28) == 1)

Please choose only one of the following:

1. Yes
2. No

### [Q6] What was the date of your incorporation?

Only answer this question if the following conditions are met: ([Q4](file:///C:\index.php\questionAdministration\view\surveyid\416974\gid\2\qid\28) == 2 or [Q4](file:///C:\index.php\questionAdministration\view\surveyid\416974\gid\2\qid\28) == 3)

Your answer must be between 2000 and 2022.

Only an integer value may be entered in this field.

Please write a year [_][_][_][_]

## Objectives and activities

### [Q7] Please describe the main objectives of your organisation.

Please write your answer here:

### [Q8] What are you main (and subsidiary) things that you do?

Please choose the appropriate response for each item:

|  | Main | Subsidiary |
| --- | --- | --- |
| Provide information to supporters | ⭘ | ⭘ |
| Media – that is, contacts with journalists | ⭘ | ⭘ |
| Raise profile of Safer Nicotine Products on social media | ⭘ | ⭘ |
| Discuss regulatory and policy issues on social media | ⭘ | ⭘ |
| Produce materials for broadcast streaming in audio or video | ⭘ | ⭘ |
| Run campaigns | ⭘ | ⭘ |
| Make contact with parliamentarians/government officials | ⭘ | ⭘ |
| Make and maintain international contacts | ⭘ | ⭘ |
| Organize meetings | ⭘ | ⭘ |
| Provide information such as newsletters and or websites | ⭘ | ⭘ |
| Responding to consultations | ⭘ | ⭘ |

### [Q8a] Other, please specify

Please write your answer here:

### [Q9] Compared to 12 months ago, how would you rate the change in the dynamics of your organisation?

Please choose only one of the following:

1. We now engage in many more activities than we did 12 months ago
2. We now engage in more activities than we did 12 months ago
3. The activity of our organization remains the same
4. We engage in less activity now than we did 12 months ago
5. We now engage in far less activity than we did 12 months ago

## Products and geography

### [Q10] What best describes the safer nicotine products do you advocate for?

Please choose all that apply:

1. E-cigarettes – we are mainly a vaping organisation
2. Snus – we mainly advocate for snus
3. Nicotine pouches – we mainly advocate for nicotine pouches
4. Heated tobacco products – we mainly advocate for HTP
5. Nicotine replacement therapy - we mainly advocate for NRT
6. Other safer nicotine products, please specify below
7. All/most safer nicotine products, without any special focus

### [Q10a] Please indicate which other safer nicotine products your organisation advocates for?

Only answer this question if the following conditions are met: ([Q10_SQ007](file:///C:\index.php\questionAdministration\view\surveyid\416974\gid\4\qid\46) == 'Y')

Please write your answer here:

### [Q11] Where does your organisation operate?

Please choose only one of the following:

1. It operates in a region or state within a country.
2. It is national, it works across the whole or most of the country.
3. It is regional, it works within a specified geographical region.
4. It is international, it works across several countries.

### [Q11a] Comments that will help us understand where you operate

Please write your answer here:

### [Q12] In which country or countries do you mainly work?

Please write your answer here:

### [Q13] In which language(s) do you work?

Please write your answer here:

## Structure and membership

### [Q14] Which of the following best describes how your organisation is run.

Please choose only one of the following:

1. We have no formal organisational structure.
2. We have an informal structure.
3. We have a formal governing board, for example a board of trustees, directors, or company owners.

### [Q15] Do you have formal members, that is, people who have joined the organisation and are on a membership, supporters or associates list? If so, how many members do you have?

Your answer must be at least 0.

Only an integer value may be entered in this field.

Please write your answer here:

### [Q15a] Comment if needed, for example, different types of membership.

Please write your answer here:

### [Q16] Has your organisation's membership decreased, increased, or stayed the same over the past 12 months?

Please choose only one of the following:

1. Our membership has increased
2. Our membership remains unchanged
3. Our membership has decreased

## Internet presence

### [Q17] Is your organisation present on the Internet?

Please choose only one of the following:

1. Yes
2. No

### [Q18] What type of Internet presence does your organisation have?

Only answer this question if the following conditions are met: ([Q17](file:///C:\index.php\questionAdministration\view\surveyid\416974\gid\6\qid\85) == 1))

Comment only when you choose an answer.

Please choose all that apply and provide a comment:

1. WWW page, please enter address
2. Facebook group, please enter name
3. Facebook page, please enter name
4. Instagram account, please enter name
5. Twitter account, please enter name
6. Profile or/and group or/and page on other social media, please specify
7. Communicator group (Whatsapp, Messenger, Signal, Skype, etc.)
8. Streaming channel, for example YouTube, Vimeo, audio podcasts, please specify
9. Forum, discussion or chat list
10. Other, please specify

### [Q19] How many people does your organisation reach?

Only an integer value may be entered in this field.

Please write your answer here:

### [Q19a] Comment

Please write your answer here:

## Staff

### [Q20] Does your organisation employ or contract any paid staff?

Please choose only one of the following:

1. Yes
2. No

### [Q21] If yes, could you please list their roles and average working hours per week.

Only answer this question if the following conditions are met: ([Q20](file:///C:\index.php\questionAdministration\view\surveyid\416974\gid\7\qid\131) == 1)

Please fill in from 1 to 20 answers.

Please write one role per line. Enter average working hours per week after comma.

### [Q21a] Comment

Only answer this question if the following conditions are met: ([Q20](file:///C:\index.php\questionAdministration\view\surveyid\416974\gid\7\qid\131) == 1)

Please write your answer here:

### [Q22] What is the size of your core team?

Only an integer value may be entered in this field.

By ‘core team’ we mean the total number of people who do most of the work.

These can be paid or unpaid.

Please write the approximate number of all.

Please write your answer here:

## Funding

### [Q23] Is your organisation open to accepting industry funding?

Please choose only one of the following:

1. Yes
2. No

### [Q24] Does your organisation have any funding?

Please choose only one of the following:

1. Yes
2. No

### [Q24a] Yes, we have funding from:

Only answer this question if the following conditions are met: ([Q24](file:///C:\index.php\questionAdministration\view\surveyid\416974\gid\8\qid\172) == 1)

Please select at least one answer.

Please choose all that apply:

1. Member contributions for example annual membership fees
2. Grants
3. Donations
4. Funding from foundations
5. Funding from government
6. Funding in kind or ‘pro bono’
7. Funding from vaping companies
8. Funding from pharmaceutical companies
9. Funding from tobacco companies
10. Other

### [Q24b] Please describe your grants.

Only answer this question if the following conditions are met: (Q24a_SQ2 == 1)

Please write your answer here:

### [Q25] What is your organisation's annual income?

Only an integer value may be entered in this field.

Please write below an estimate value in U$D of your funds received for the last full year.

Please write your answer here:

## Achievements, obstacles, challenges

### [Q26] What are your organisation's main achievements in the last 12 months?

Please write your answer here:

### [Q27] What are the main obstacles that you face as an organisation?

Please write your answer here:

### [Q28] What are the main challenges regarding safer nicotine products in your country in the next 12 months?

Please choose all that apply:

1. Potential sale bans
2. Potential taxation
3. Potential restrictions (flavour bans, nicotine content etc.)
4. Potential bans on using SNP in public places
5. Other

### [Q29] What do you need to operate more effectively?

Please choose all that apply:

1. Funding
2. Paid staff / more paid staff
3. More volunteers
4. Skills training – please state what (eg media relations, social media, finance, fund raising)
5. Support from and links with other advocacy groups
6. Resources, such as briefing papers, documents
7. Own language resources
8. Other

### [Q29a] Please state what skills training (e.g. media relations, social media, finance, fund-raising)

Only answer this question if the following conditions are met: ([Q29_SQ4](file:///C:\index.php\questionAdministration\view\surveyid\416974\gid\9\qid\287) == 1)

Please write your answer here:

## End notes

### [Q30] Any other comments?

Please write your answer here:

Thank you for completing this survey.

If you have any question, please ask {ADMINEMAIL}.
