# Supplementary material for: Global survey of consumer organizations advocating for safer nicotine products
Source: Public Health Chall. 2023 Jan 19;2(1):e58. doi: 10.1002/puh2.58 (PMC12039648; doi:10.1002/puh2.58)
Supplement: Supplementary file 2 — Invitation Letter [file PUH2-2-e58-s001.docx]

Dear Sir or Madam,

We need your help with the first-ever global survey of nicotine consumer organisations.

These organisations have been hugely significant in raising the profile of safer nicotine products and advocating for them nationally and globally. However, there is much misinformation about these organisations and how they operate. What little has been written has mainly been negative and misunderstands their nature, role, resources and funding.

The Global State of Tobacco Harm Reduction group is therefore attempting to map the extent of consumer advocacy organisations globally, and to find out more about how they work, their resources, aims, and the obstacles they have encountered.

The aim of this study is to:

- map the number and location of nicotine consumer organisations globally, regionally and nationally, and by language
- describe their history, legal status, membership, structure, objectives, working methods and activities, and funding
- describe different types of organisations
- report achievements, impacts and obstacles.

**Our questionnaire**

We would very much appreciate your help.

We have a short online questionnaire which needs to be completed on behalf of your organisation, and we will approach some people for a more in-depth interview conducted by email or by Zoom/Skype.

The survey asks questions about the history, structure, membership, organisation, resources, funding and activities of your organisation.

**Can your organisation participate?**

To be included in the study your organisation must:

- be concerned with safer nicotine products i.e. non-combustible products such as e-cigarettes, Swedish style snus or nicotine pouches, heated tobacco products
- have a within country, national, regional or international focus
- undertake advocacy, such as by using media and social media, organising meetings, contacting parliamentarians
- not be primarily a trade or product association, though hybrid organisations are eligible

You do not need to be a legally recognized organisation.

**Ethics and data protection**

The research has received ethics permission from the University of Warsaw.

Participation is voluntary and there is no payment for participation. Questions refer to the organisation and no personal information is collected.

No personal information will be stored except names and email addresses for contact and survey purposes.

Individuals will not be identifiable in any publication. Many groups are small and often run by one or two people, and names of organisations will only be included in any publications or report with further consent. This will avoid the possibility that an individual might be identified through the name of the organisation and its country.

The raw data set will not be publicly available or accessible. Information will be stored securely with access only by the investigators and access protected by two-stage verification. Respondents’ names and contact details will be kept separate from the main dataset and linked only by code number.

**Who should complete the questionnaire?**

If you think you are eligible and would like to help please let us know if you are the best person to answer on behalf of your organisation or whether there is someone else in your group who should do this.

If your organisation can help us we will then send you a unique code to do the online survey.

If you have any questions or concerns please contact Tomasz Jerzyński [tom@kachange.eu](mailto:tom@kachange.eu), Gerry Stimson [gerry@kachange.eu](mailto:gerry@kachange.eu), or Jessica Harding [jessica@kachange.eu](mailto:jessica@kachange.eu).

With kind regards

Gerry Stimson

Jess Harding

Tom Jerzynski

***About us:*** [*Knowledge•Action•Change*](https://kachange.eu/)*(K•A•C) promotes harm reduction as a key public health strategy grounded in human rights. The team has over forty years of experience of harm reduction work in drug use, HIV, smoking, sexual health, and prisons. K•A•C runs the*[*Global State of Tobacco Harm Reduction (GSTHR)*](https://gsthr.org/) *which maps the development of tobacco harm reduction and the use, availability and regulatory responses to safer nicotine products, as well as smoking prevalence and related mortality, in over 200 countries and regions around the world. For all publications and live data, visit* [*https://gsthr.org*](https://gsthr.org)

***Our funding****: The GSTHR project is produced with the help of a grant from the* [*Foundation for a Smoke Free World*](http://smokefreeworld.org/)*, an independent, US non-profit 501(c)(3) which, under US law, must operate independently of its donors. The project and its outputs are, under the terms of the grant agreement, independent of the Foundation.*
